# Hue-Preserving Saturation Improvement in RGB Color Cube

**DOI:** 10.3390/jimaging7080150

**Published:** 2021-08-18

**Authors:** Kohei Inoue, Minyao Jiang, Kenji Hara

**Affiliations:** Department of Communication Design Science, Faculty of Design, Kyushu University, 4-9-1, Shiobaru, Minami-ku, Fukuoka 815-8540, Japan; jminyao97@gmail.com (M.J.); hara@design.kyushu-u.ac.jp (K.H.)

**Keywords:** color image enhancement, hue preservation, saturation improvement, gamut problem, histogram equalization, histogram specification

## Abstract

This paper proposes a method for improving saturation in the context of hue-preserving color image enhancement. The proposed method handles colors in an RGB color space, which has the form of a cube, and enhances the contrast of a given image by histogram manipulation, such as histogram equalization and histogram specification, of the intensity image. Then, the color corresponding to a target intensity is determined in a hue-preserving manner, where a gamut problem should be taken into account. We first project any color onto a surface in the RGB color space, which bisects the RGB color cube, to increase the saturation without a gamut problem. Then, we adjust the intensity of the saturation-enhanced color to the target intensity given by the histogram manipulation. The experimental results demonstrate that the proposed method achieves higher saturation than that given by related methods for hue-preserving color image enhancement.

## 1. Introduction

Image enhancement is the process of the quality improvement of an image for both human viewers and other automated image processing techniques [1]. Although there are many books that discuss the techniques for image enhancement for grayscale images, the generalization of these techniques to color images is not straightforward because color images have more factors to be considered than grayscale images [2], and those factors, e.g., hue, saturation and lightness, are mutually dependent on each other. This situation makes it more challenging to enhance color images than grayscale ones.

The performance evaluation of image-enhancement techniques has also been the main topic in the field of image processing research and development. For example, Puniani and Arora used two quantitative measures: the contrast improvement index (CII) and Tenengrad measure [3], both of which focus on the local contrast of target images. However, the quality of color images is not evaluated by contrast only. Huang et al. [4] used two color image quality metrics—the color naturalness index (CNI) and color colorfulness index (CCI), based on luminance, hue and saturation—to demonstrate that the closer CNI is to 1 in the range [0,1], the more natural the color of an image, and the best range for CCI is between 16 and 20, according to [5]. These results show the mutual dependence of color attributes, and the relationship between saturation and colorfulness. When the luminance or intensity is predetermined by an appropriate contrast enhancement technique, which is the case that we discuss in this paper, we can improve the colorfulness by increasing saturation while preserving hue.

Color image enhancement is one of the main issues in digital image processing for human visual systems and computer vision. A color has three attributes, including hue, saturation and intensity (or lightness), which are modified by color image enhancement. Among the three attributes of a color, hue determines the appearance of the color. Therefore, it is often preferable to preserve hue in the process of color image enhancement. A typical way of preserving hue is color space conversion, i.e., we convert an RGB (red, green and blue) color, which is a typical format for storing colors in computers, into the HSI (hue, saturation and intensity) color, then modify saturation and intensity, and finally return the modified HSI color to the original RGB color space. However, such a naive procedure may cause a gamut problem in that the modified color may be beyond the color gamut. A gamut problem, which is also called the out-of-gamut problem [6], is a common problem in color image processing, where color space transformations between an RGB color space and other color spaces compatible with human perception are utilized. Different color spaces or color coordinate systems have a different color gamut, and color image processing in each color space is independent of other color spaces. Therefore, the domain and range of gamut mapping do not match generally, which may influence the results of color image processing, e.g., Yang and Kwok proposed a gamut clipping method for avoiding such out-of-gamut problems [6]. However, clipping any value of color attributes may cause the degeneration of different colors into the same color.

To overcome the gamut problem, Naik and Murthy proposed a scheme for hue-preserving color image enhancement without a gamut problem [2], which was surveyed by Bisla [7]. Han et al. also presented an equivalent method to theirs from the point of view of 3D color histogram equalization [8]. However, as they pointed out, their scheme always decreases the saturation in a common condition [2]. To alleviate such a decrease in saturation, Yang and Lee proposed a modified hue-preserving gamut mapping method, which outputs the color with a higher saturation than that given by Naik and Murthy’s method [9]. Yang and Lee’s method partitions the range of luminance, which is a normalized intensity, into three parts, corresponding to dark, middle and bright colors. For a dark or bright color, their method first enhances the saturation, and then applies Naik and Murthy’s method to the enhanced color. However, for a color with the luminance in the middle range, Yang and Lee’s method is the same as that of Naik and Murthy. Therefore, we cannot improve the saturation of such colors, even if we adopt the method of Yang and Lee. Yu et al. proposed a method for improving the saturation, using three bisecting planes of an RGB color cube, and demonstrated that their method achieves higher saturation, compared with the methods of Naik and Murthy, and Yang and Lee [10]. Park and Lee proposed a piecewise linear gamut mapping method, which balances color saturation while avoiding color clipping [11].

In this paper, we propose a method for hue-preserving saturation improvement by eliminating the middle part, where Yang and Lee’s method is the same as that of Naik and Murthy, which decreases the saturation. As a result, we can minimize the decrease in saturation caused by hue-preserving color image enhancement. We assume that the hue, saturation and intensity are defined in the HSI color model [12]. We first compute the target intensities for all pixels by histogram equalization (HE) [13] or histogram specification (HS), which is also called histogram matching [14], where an RGB color cube-based method [15] is adopted for further improvement of saturation. Then, we project each color in a given input image onto a surface in an RGB color space, which bisects the RGB color cube into dark and bright parts. The surface spreads to the edges of the RGB color cube far from the center of the cube, which denotes a neutral gray point. Therefore, the projection of an RGB color point onto the surface can increase the saturation. After that, we move the saturation-improved color to the final color point, corresponding to the target intensity. In our experiment, we compare the proposed method with those of Naik and Murthy, and Yang and Lee, and demonstrate the effectiveness of the proposed method.

In this work, we address the research question of how to maximize the color saturation in the context of hue-preserving color image enhancement. The reason why we want to preserve the original hue is that we want to keep the image contents unchanged, before and after the process of color image enhancement. For example, if red becomes green by a hue-changing color image enhancement, then a red apple will become a green one, which will alter the appearance of the image greatly. In order to keep the appearance unchanged, we consider it important for the color image enhancement to preserve the hue. Previous works had subregions in an RGB color space in which the colors always fade by the enhancement procedure. To avoid the effect of color fading, we try to eliminate the subregion for maximizing the effect of the saturation improvement. This elimination of the subregion is our main contribution.

The rest of this paper is organized as follows: Section 2 summarizes the histogram manipulation methods used in the following sections. Section 3 summarizes Naik and Murthy’s method and that of Yang and Lee for hue-preserving color image enhancement, and proposes a method for improving the saturation of Yang and Lee’s method by avoiding the direct use of Naik and Murthy’s method in the middle range of the intensity axis. Section 4 shows our experimental results, which is discussed in Section 5. Finally, Section 6 concludes this paper.

## 2. Histogram Manipulation

Let $P=[p_{ij}]$ be an RGB (red, green and blue) color image for i=1,2,…,m and j=1,2,…,n, where $p_{ij}={\left[r_{ij},g_{ij},b_{ij}\right]}^{T}$ denotes the RGB color vector of a pixel at the two-dimensional point (i,j) on the image *P* with *m* rows and *n* columns of pixels, and the superscript *T* denotes the matrix transpose. Assume that *P* is a 24-bit true color image; then, every element of $p_{ij}$ is an integer between 0 and 255 such that $r_{ij}\in \left\{0,1,\ldots ,255\right\}$, i.e., we equally assign 8-bit to each element to express $2^{8}=256$ levels. The intensity of $p_{ij}$ is defined by $l_{ij}=r_{ij}+g_{ij}+b_{ij}$, from which we have $l_{ij}\in \left\{0,1,\ldots ,L\right\}$ for L=255×3=765. Instead of the intensity, LHS (luminance, hue and saturation) and YIQ (Luminance (Y), In-phase Quadrature in NTSC (National Television System Committee) color space) color spaces define the luminance *L* or the Y component *Y*: L=Y=0.299R+0.587G+0.114B for a normalized RGB color vector ${\left[R,G,B\right]}^{T}$ with 0≤R,G,B≤1. Both of them can be expressed as the inner product of a constant vector and an RGB color vector, and are proportional to the length of the RGB color vector. Therefore, they have essentially equivalent information.

In this section, we briefly summarize two histogram manipulation (HM) methods: histogram equalization (HE) and histogram specification (HS).

### 2.1. Histogram Equalization

Histogram equalization (HE) is an effective method for contrast enhancement as used by Naik and Murthy [2]. The histogram of the intensity image of *P* can be equalized as follows. Let $h=[h_{0},h_{1},\ldots ,h_{L}]$ be the histogram; then, the *l*th element of h is given by $h_{l}=\sum_{i=1}^{m}\sum_{j=1}^{n}\delta_{l,l_{ij}}$, where $\delta_{l,l_{ij}}$ denotes the Kronecker delta defined by $\delta_{l,l_{ij}}=1$ if $l=l_{ij}$, and 0 if $l\neq l_{ij}$. Next, let $h=[H_{0},H_{1},\ldots ,H_{L}]$ be the cumulative histogram of h; then, the *l*th element of h is given by $H_{l}=\sum_{k=0}^{l}h_{k}$. Then, HE transforms an intensity $l_{ij}$ into the following:

(1) $$\begin{array}{c} {\tilde{l}}_{ij}=\mathrm{round}\left(L\frac{H_{l_{ij}}}{H_{L}}\right), \end{array}$$

where the ‘round’ operator rounds a given argument toward the nearest integer, and $H_{L}=\sum_{k=0}^{L}h_{k}=\sum_{k=0}^{L}\sum_{i=1}^{m}\sum_{j=1}^{n}\delta_{k,l_{ij}}=\sum_{i=1}^{m}\sum_{j=1}^{n}1=mn$.

### 2.2. Histogram Specification

Histogram specification (HS) assumes that a target histogram is given, and transforms the original histogram into the target one. Let $\tilde{h}=\left[{\tilde{h}}_{0},{\tilde{h}}_{1},\ldots ,{\tilde{h}}_{L}\right]$ be a target histogram into which we want to transform the original histogram of intensity, and let $\tilde{H}=\left[{\tilde{H}}_{0},{\tilde{H}}_{1},\ldots ,{\tilde{H}}_{L}\right]$ be the cumulative histogram of $\tilde{h}$, i.e., the $\tilde{l}$th element of $\tilde{h}$ is given by ${\tilde{H}}_{\tilde{l}}=\sum_{k=0}^{\tilde{l}}{\tilde{h}}_{k}$. Then, HS transforms an intensity $l_{ij}$ into the following:

(2) $$\begin{array}{c} {\tilde{l}}_{ij}=\arg\min_{\tilde{l}\in \left\{0,1,\ldots ,L\right\}}\left\{\left|H_{L}{\tilde{H}}_{\tilde{l}}-{\tilde{H}}_{L}H_{l_{ij}}\right|\right\}. \end{array}$$

As a reasonable choice for the target histogram, we have proposed an RGB color cube-based method as follows [15]: We compute the area of the cross section of a normalized RGB color cube ${\left[0,\;1\right]}^{3}$ and an equi-intensity plane $1^{T}p=∥1∥l$, where $1={\left[1,1,1\right]}^{T}$, $p={\left[r,g,b\right]}^{T}\in {\left[0,\;1\right]}^{3}$, and ∥·∥ denotes the Euclidean norm, as a function of l∈[0,3]:

(3) $$\begin{array}{c} a\left(l\right)=\left\{\begin{array}{cc} a_{1}\left(l\right)=\frac{\sqrt{3}}{2}l^{2} & \mathrm{if}\;0\leq l\leq 1,\\ a_{2}\left(l\right)=\frac{3\sqrt{3}}{4}-\sqrt{3}{\left(l-\frac{3}{2}\right)}^{2} & \mathrm{if}\;1\leq l\leq 2,\\ a_{3}\left(l\right)=\frac{\sqrt{3}}{2}{\left(3-l\right)}^{2} & \mathrm{if}\;2\leq l\leq 3, \end{array}\right. \end{array}$$

from which the target histogram is given by the following:

(4) $$\begin{array}{c} {\tilde{h}}_{\tilde{l}}=a\left(\frac{3\tilde{l}}{L}\right) \end{array}$$

for $\tilde{l}=0,1,\ldots ,L$. We also have the integral of a(l) as follows:

(5) $$\begin{array}{cc} A(l) & =\int_{0}^{l}a\left(x\right)dx\\ \; & =\left\{\begin{array}{cc} A_{1}\left(l\right)=\int_{0}^{l}a_{1}\left(x\right)dx=\frac{\sqrt{3}}{6}l^{3} & \mathrm{if}\;0\leq l\leq 1,\\ A_{2}\left(l\right)=A_{1}\left(1\right)+\int_{1}^{l}a_{2}\left(x\right)dx=\frac{\sqrt{3}}{6}\left(3-2l^{3}+9l^{2}-9l\right) & \mathrm{if}\;1\leq l\leq 2,\\ A_{3}\left(l\right)=A_{2}\left(2\right)+\int_{2}^{l}a_{3}\left(x\right)dx=\frac{\sqrt{3}}{6}\left[6-{\left(3-l\right)}^{3}\right] & \mathrm{if}\;2\leq l\leq 3, \end{array}\right. \end{array}$$

from which the target cumulative histogram is given by the following:

(6) $$\begin{array}{c} {\tilde{H}}_{\tilde{l}}=A\left(\frac{3\tilde{l}}{L}\right) \end{array}$$

for $\tilde{l}=0,1,\ldots ,L$. This method regards the area of the cross section between the color cube and a plane perpendicular to the intensity axis as the value of the target histogram. Since the wider the cross section becomes, the more saturated colors it contains, it is expected that HS with the obtained target histogram can produce saturation-enhanced images.

**Figure 1:**
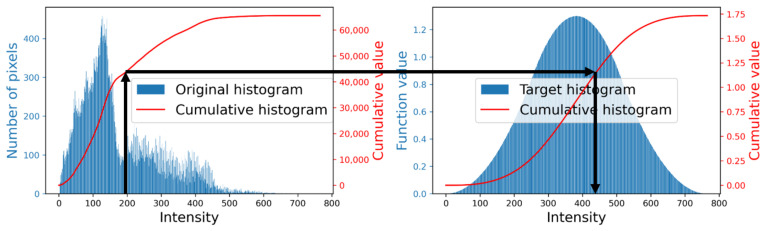
The left and right graphs show the original and target histograms with blue bars, and their cumulative counterparts are also shown in red curves. An input intensity is transformed into the output one as indicated by black arrows.

**Figure 2:**
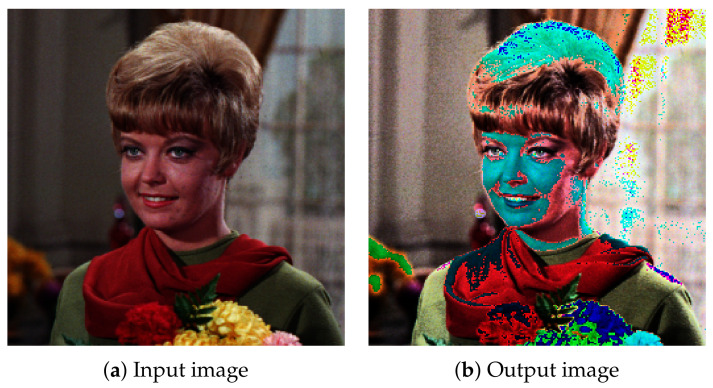
Color image enhancement by histogram equalization with the gamut problem. The intensity histogram of an input color image (**a**) is equalized, and the corresponding colors in the output image (**b**) are computed by (7). The computed color values are saved with the ‘ Image.save’ function in Python Image Library (PIL).

Figure 1 illustrates the procedure of HS based on this method. In this figure, the left graph shows an original intensity histogram (the blue bars) of a color image shown in Figure 2a and its cumulative version (the red curve), and the right graph shows the target histogram (the blue bars) in (4) and its cumulative version (the red curve) in (6). We first extend an arrow upward from a point on the horizontal axis of the left graph, which denotes an input intensity, to the red curve. Then we turn right, and move to the right graph to find the intersection point with another red curve. Finally, we go down from the intersection point to the horizontal axis of the right graph, on which the indicated point gives the output intensity.

**Figure 3:**
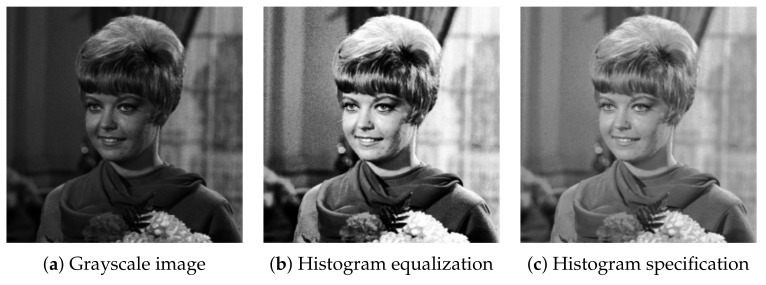
Histogram manipulation: the contrast of an input grayscale image (**a**), which is an image in the SIDBA [16] image dataset, is enhanced by histogram equalization (**b**) and RGB color cube-based histogram specification (**c**).

Figure 3 shows an example of HM for a grayscale image, where the original image in Figure 3a, which is in the standard image database (SIDBA) [16], is slightly dark. HE in Figure 3b enhances the contrast; however, an excessive enhancement makes some details invisible, e.g., a leaf on the girl’s chest is assimilated into her scarf. On the other hand, HS in Figure 3c enhances the overall details moderately.

## 3. Hue-Preserving Color Image Enhancement

A straightforward way of applying HM of an intensity image to the corresponding color image may be to rescale all colors to adjust their intensities to targets as follows:

(7) $$\begin{array}{c} {\tilde{p}}_{ij}=\frac{{\tilde{l}}_{ij}}{l_{ij}}p_{ij}, \end{array}$$

where $l_{ij}$ and ${\tilde{l}}_{ij}$ denote the original and target intensities, respectively. However, this may cause a gamut problem when ${\tilde{p}}_{ij}$ goes out of the range of color gamut. For example, a color image in Figure 2a is broken by the method in (7) with ${\tilde{l}}_{ij}$ computed by HE as shown in Figure 2b, where the computed color values are simply saved with the ‘Image.save’ function in Python Image Library (PIL).

### 3.1. Naik and Murthy’s Method

To avoid the above gamut problem, Naik and Murthy proposed a method that ensures that the modified colors always go into the color gamut as follows [2]: Let $p={\left[r,g,b\right]}^{T}\in {\left[0,\;1\right]}^{3}$ be an RGB color vector in a normalized RGB color cube ${\left[0,\;1\right]}^{3}$ for notational simplicity, and assume that a target intensity $\tilde{l}\in \left[0,\;3\right]$ is given. Then, we compute the intensity of p as l=r+g+b∈[0,3], and compare it with $\tilde{l}$. If $\tilde{l}\leq l$, then (7) is adopted without a gamut problem because $0\leq \frac{\tilde{l}}{l}\leq 1$ ensures that $\tilde{p}=\frac{\tilde{l}}{l}p\in {\left[0,\;1\right]}^{3}$.

On the other hand, if $\tilde{l}>l$, then (7) may cause a gamut problem. To avoid this, we transform p into the corresponding CMY (cyan, magenta and yellow) color vector as $\bar{p}=1-p$. The intensity of $\bar{p}$ is given by $\bar{l}=\left(1-r\right)+\left(1-g\right)+\left(1-b\right)=3-l$. Similarly, the target intensity in the CMY color space is given by $\bar{\tilde{l}}=3-\tilde{l}$. Then we compute the modified CMY color vector $\tilde{\bar{p}}=\frac{\bar{\tilde{l}}}{\bar{l}}\bar{p}$, which does not cause any gamut problem in the CMY color space because $\bar{l}-\bar{\tilde{l}}=\left(3-l\right)-\left(3-\tilde{l}\right)=\tilde{l}-l>0$ or $\frac{\bar{\tilde{l}}}{\bar{l}}<1$. Finally, we transform $\bar{\tilde{p}}$ back into the RGB color vector as the following:

(8) $$\begin{array}{c} \tilde{p}=1-\bar{\tilde{p}}=1-\frac{\bar{\tilde{l}}}{\bar{l}}\bar{p}=1-\frac{3-\tilde{l}}{3-l}\left(1-p\right). \end{array}$$

Naik and Murthy’s method is summarized in Algorithm 1.
**Algorithm 1** Naik and Murthy’s method**Require** **:**RGB color vector $p={\left[r,g,b\right]}^{T}\in {\left[0,\;1\right]}^{3}$, target intensity $\tilde{l}\in \left[0,\;3\right]$**Ensure** **:**Modified RGB color vector $\tilde{p}$1:Compute the intensity of p as l=r+g+b;2:**if**$\tilde{l}\leq l$**then**3: $\tilde{p}:=\frac{\tilde{l}}{l}p$;4:**else**5: $\tilde{p}:=1-\frac{3-\tilde{l}}{3-l}\left(1-p\right)$;6:**end if**

We can see that the above method does not increase the saturation defined by the following:

(9) $$\begin{array}{c} S\left(p\right)=∥\left[I-\left(\frac{1}{∥1∥}\right){\left(\frac{1}{∥1∥}\right)}^{T}\right]p∥, \end{array}$$

which is the distance from the intensity axis joining the black and white vertices to p [12], where *I* denotes the 3×3 identity matrix. We have proved that the following equations hold for any nonnegative numbers β and γ [15]:

(10) $$\begin{array}{cc} S(\beta p) & =\beta S(p) \end{array}$$

(11) $$\begin{array}{cc} S(1-\gamma (1-p)) & =\gamma S(p). \end{array}$$

The lines 3 and 5 in Algorithm 1 correspond to the cases where $\beta =\frac{\tilde{l}}{l}\leq 1$ and $\gamma =\frac{3-\tilde{l}}{3-l}<1$ in the above Equations (10) and (11), both of which mean a non-increase in saturation.

### 3.2. Yang and Lee’s Method

To increase the saturation, Yang and Lee modified Naik and Murthy’s method as follows [9]. First, we divide the normalized RGB color space into three parts by two planes perpendicular to the intensity axis: $1^{T}p=1$ and $1^{T}p=2$. If a color vector p satisfies $1^{T}p=l<1$, then the intensity of p is boosted by $p^{\prime }=p/l$ to $1^{T}p^{\prime }=1^{T}p/l=l/l=1$. After that, $p^{\prime }$ is passed to the function $\phi^{NM}$, which computes Naik and Murthy’s method in Algorithm 1 to obtain a modified color vector $\tilde{p}=\phi^{NM}\left(p^{\prime },\tilde{l}\right)$.

On the other hand, if p satisfies $1^{T}p=l>2$, then the intensity of p is dropped down to 2 to increase the saturation as follows. First, we transform p into the CMY color vector as $\bar{p}=1-p$ whose intensity is $1^{T}\bar{p}=1^{T}\left(1-p\right)=3-l<1$. Next, the intensity is boosted up by ${\bar{p}}^{\prime }=\bar{p}/\left(3-l\right)$ to $1^{T}{\bar{p}}^{\prime }=1^{T}\bar{p}/\left(3-l\right)=\left(3-l\right)/\left(3-l\right)=1$. After that, we transform ${\bar{p}}^{\prime }$ back into the RGB color vector as $p^{\prime }=1-{\bar{p}}^{\prime }=1-\bar{p}/\left(3-l\right)=1-\left(1-p\right)/\left(3-l\right)$. Finally, $p^{\prime }$ is passed to the function $\phi^{NM}$ to obtain a modified color vector $\tilde{p}=\phi^{NM}\left(p^{\prime },\tilde{l}\right)$. If 1≤l≤2, then we simply apply Naik and Murthy’s method to p as $\tilde{p}=\phi^{NM}\left(p,\tilde{l}\right)$.

Yang and Lee’s method is summarized in Algorithm 2.
**Algorithm 2** Yang and Lee’s method.**Require** **:**RGB color vector $p={\left[r,g,b\right]}^{T}\in {\left[0,\;3\right]}^{3}$, target intensity $\tilde{l}\in \left[0,\;3\right]$**Ensure** **:**Modified RGB color vector $\tilde{p}$1:Compute the intensity of p as l=r+g+b;2:**if** l<1 **then**3: $p^{\prime }:=p/l$;4: $\tilde{p}:=\phi^{NM}\left(p^{\prime },\tilde{l}\right)$;5:**else if** l>2 **then**6: $p^{\prime }:=1-\frac{1}{3-l}\left(1-p\right)$;7: $\tilde{p}:=\phi^{NM}\left(p^{\prime },\tilde{l}\right)$;8:**else**9: $\tilde{p}:=\phi^{NM}\left(p,\tilde{l}\right)$;10:**end if**

We have proved that the saturation given by Yang and Lee’s method is greater than or equal to that given by Naik and Murthy’s method [15]. However, as described on line 9 in Algorithm 2, Yang and Lee’s method directly uses Naik and Murthy’s method for 1≤l≤2. Therefore, Yang and Lee’s method also does not increase the saturation of any color whose intensity falls in the range.

Regarding the drawback of Yang and Lee’s method, Park and Lee recently proposed a piecewise linear gamut mapping method, which employs multiplicative and additive color mapping to improve the color saturation [11]. However, as pointed out by the authors, the color shifting in the additive algorithm used in the middle part may cause a gamut problem, rarely. Therefore, they added an exceptional procedure to avoid the problem. In the next subsection, we consider a gamut problem-free method.

### 3.3. Middle Part Elimination

In this subsection, we propose a method for eliminating the middle part in Yang and Lee’s method, where Naik and Murthy’s method is directly used without boosting the saturation of colors. More precisely, Yang and Lee’s method divides the RGB color space into three disjoint parts with two planes perpendicular to the intensity axis *l*: l<1, 1≤l≤2 and 2<l; in the middle part, satisfying 1≤l≤2, their method cannot increase the saturation as well as Naik and Murthy’s method. On the other hand, we divide the RGB color space into two parts with a boundary surface: l≤θ and θ<l, where θ denotes the boundary threshold. We refer to this change from tripartition to bipartition as the middle part elimination.

The method described in this subsection is a refined version of Yu’s method [10], which uses three planes for bisecting an RGB color cube. Those planes have unused regions redundantly, which are removed in the refined method. Moreover, the algorithm is simplified as described in Algorithm 3.

**Figure 4:**
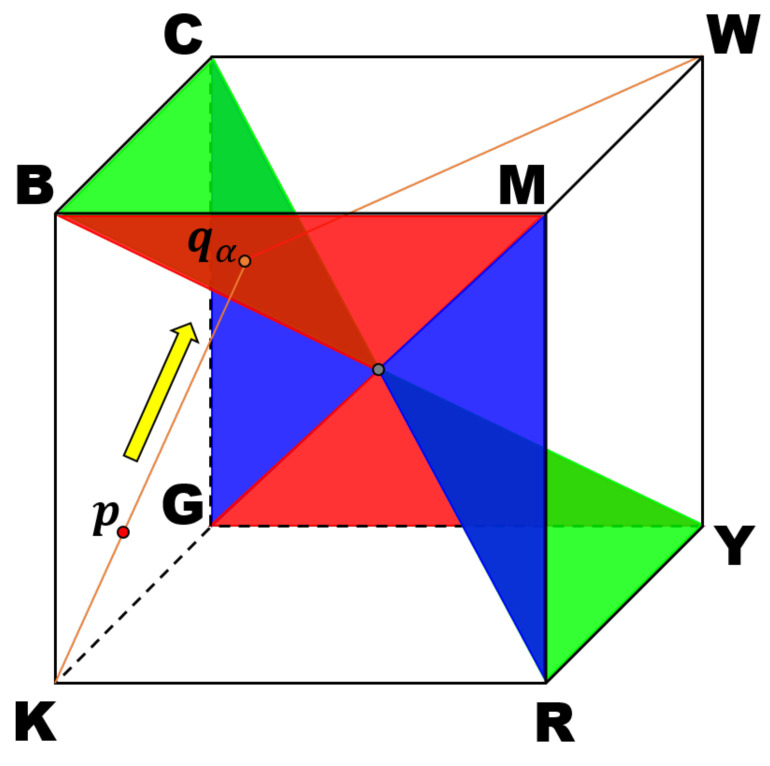
RGB color space, illustrated by a cube is bisected by a boundary surface, which passes through the center of the space. The letters attached to the vertices of the cube denote eight basic colors, normalized as follows: $K={\left[0,0,0\right]}^{T},\;R={\left[1,0,0\right]}^{T},\;G={\left[0,1,0\right]}^{T},\;B={\left[0,0,1\right]}^{T}$, $C={\left[0,1,1\right]}^{T},\;M={\left[1,0,1\right]}^{T},\;Y={\left[1,1,0\right]}^{T}$ and $W={\left[1,1,1\right]}^{T}$. Every color point in the space can be projected onto the surface to increase the saturation.

Figure 4 shows the proposed boundary surface, which bisects the RGB color space, illustrated by a cube, into black-leaning and white-leaning parts. The letters attached to the vertices of the cube denote eight basic colors normalized as follows: $K={\left[0,0,0\right]}^{T}$, $R={\left[1,0,0\right]}^{T},\;G={\left[0,1,0\right]}^{T},\;B={\left[0,0,1\right]}^{T},\;C={\left[0,1,1\right]}^{T},\;M={\left[1,0,1\right]}^{T},\;Y={\left[1,1,0\right]}^{T}$ and $W={\left[1,1,1\right]}^{T}$. The boundary surface is painted in red, green and blue based on the coplanarity of each color point. Unlike Yang and Lee’s division of the RGB color space into three parts, the proposed bisection eliminates the middle part where Naik and Murthy’s method is directly used. Therefore, the drawback of their methods will be successfully avoided in the proposed method. For example, a color point p in this figure is first projected onto the boundary surface along the direction of the yellow arrow, where the end point is denoted by $q_{\alpha }$. Note that $q_{\alpha }$ is more distant from the intensity axis than p, which means that $q_{\alpha }$ has higher saturation than p, i.e., by projecting a color point onto the boundary surface as shown in Figure 4, we can always increase the saturation.

**Figure 5:**
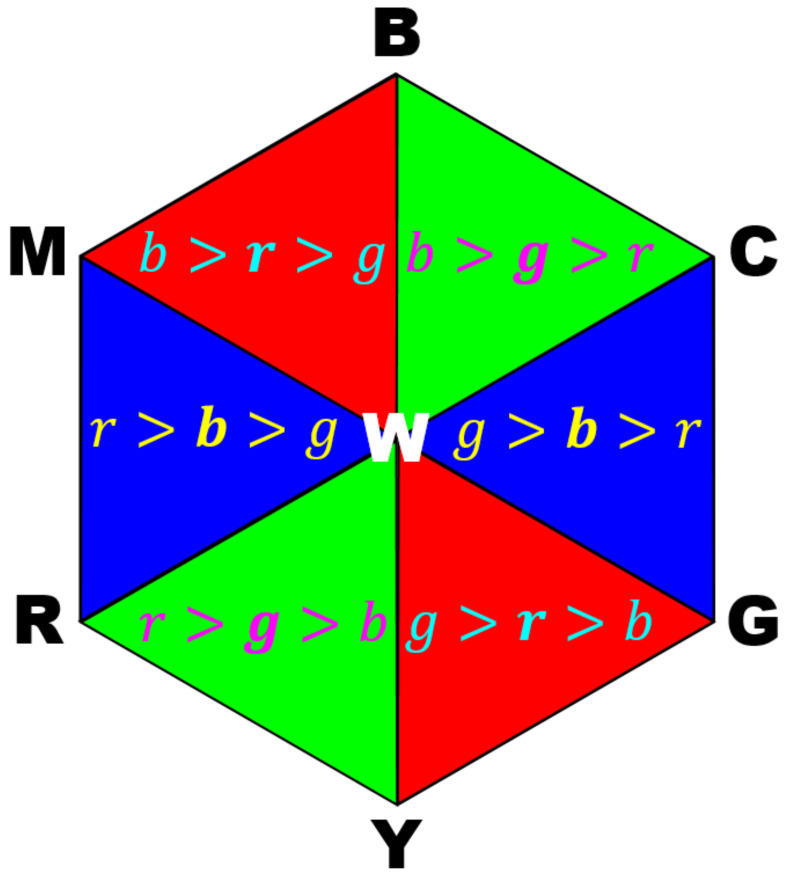
The proposed boundary surface in RGB color space viewed from the white point makes a regular hexagon, which is composed of six regular triangles painted in red, green and blue, separately. A color $p={\left[r,g,b\right]}^{T}$ falls in one of the triangles, and satisfies the inequality written in the corresponding triangle. For example, if p is orthographically projected onto the top-left red triangle BMW, then we have b>r>g.

Figure 5 shows an orthographic view of the boundary surface in Figure 4 from the white point to the black pointhidden behind the boundary surface, which forms a regular hexagon composed of six regular triangles colored in red, green and blue that correspond to the colors in Figure 4. The sides of those triangles correspond to the edges of the cube in Figure 4. A color $p={\left[r,g,b\right]}^{T}$ satisfies one of the inequalities written in the triangles onto which p is also orthographically projected. We can see that the median value of the RGB components in each triangle corresponds to the color of the triangle. This correspondence gives us an idea that elongates an RGB color vector p to the boundary surface shown in Figure 4 to increase the saturation prior to the application of Naik and Murthy’s method, i.e., we can select a plane onto which a color is projected, according to the median value of the RGB components.

In Figure 4, each pair of triangles with the same color is coplanar, where the normal vectors of red, green and blue planes are given by $u_{r}={\left[0,1,1\right]}^{T},\;u_{g}={\left[1,0,1\right]}^{T}$ and $u_{b}={\left[1,1,0\right]}^{T}$, respectively. For a color $p={\left[r,g,b\right]}^{T}$, we first select the median value in {r,g,b} as follows:

(12) $$\begin{array}{c} p_{\mathrm{med}}=\mathrm{median}\left\{r,g,b\right\}, \end{array}$$

where the ‘median’ operator selects the median value from the given values. Then, we project p onto the plane with the normal vector $u_{p_{\mathrm{med}}}$ to increase the saturation. The detailed procedure is as follows. The procedure is divided into two parts according to the position of p in the RGB color space separated by the boundary surface.

1. If $u_{p_{\mathrm{med}}}^{T}p\leq 1$, then we compute the intersection point of a line q=αp, where α is a parameter, and a plane $u_{p_{\mathrm{med}}}^{T}q=1$. From these equations, we have $\alpha =\frac{1}{l-p_{\mathrm{med}}}$ where l=r+g+b, that is, the intersection point is given by $q_{\alpha }=\frac{1}{l-p_{\mathrm{med}}}p$. Then, we apply Naik and Murthy’s method to obtain the modified color as $\tilde{p}=\phi^{NM}\left(q_{\alpha },\tilde{l}\right)$.
2. On the other hand, if $u_{p_{\mathrm{med}}}^{T}p<1$, then we compute the intersection point of a line passing through p and 1, which is expressed as q=1+α(p−1), and a plane $u_{p_{\mathrm{med}}}^{T}q=1$. From these equations, we have $\alpha =\frac{1}{2-(l-p_{\mathrm{med}})}$, that is, the intersection point is given by $q_{\alpha }=1+\frac{1}{2-(l-p_{\mathrm{med}})}\left(p-1\right)$. Then, we apply Naik and Murthy’s method to obtain the modified color as $\tilde{p}=\phi^{NM}\left(q_{\alpha },\tilde{l}\right)$.

This method is summarized in Algorithm 3.

In this algorithm, before applying Naik and Murthy’s method at line 9, every color p is modified to $q_{\alpha }$ so that the middle part where Naik and Murthy’s method is directly applied to p is eliminated. We can confirm that the saturation of $q_{\alpha }$ is not smaller than that of p as follows. Line 5 in Algorithm 3 corresponds to $\beta =\frac{1}{l-p_{\mathrm{med}}}$ in (10), and the denominator is not greater than 1 as $l-p_{\mathrm{med}}=u_{p_{\mathrm{med}}}^{T}p\leq 1$, which means that β≥1. Therefore, we find that $S\left(q_{\alpha }\right)=\beta S\left(p\right)\geq S\left(p\right)$ by (10). On the other hand, line 7 in Algorithm 3 corresponds to $\gamma =\frac{1}{2-(l-p_{\mathrm{med}})}$ in (), and the denominator is smaller than 1 as $2-\left(l-p_{\mathrm{med}}\right)=2-u_{p_{\mathrm{med}}}^{T}p<2-1=1$, which means that γ>1. Therefore, we conclude that $S\left(q_{\alpha }\right)=\gamma S\left(p\right)>S\left(p\right)$ by (11).
**Algorithm 3** The proposed method.**Require** **:**RGB color vector $p={\left[r,g,b\right]}^{T}\in {\left[0,\;3\right]}^{T}$, target intensity $\tilde{l}\in \left[0,\;3\right]$**Ensure** **:**Modified RGB color vector $\tilde{p}$1:Compute the intensity of p as l=r+g+b;2:$u_{r}:={\left[0,1,1\right]}^{T},\;u_{g}:={\left[1,0,1\right]}^{T},\;u_{b}:={\left[1,1,0\right]}^{T}$;3:Compute $p_{\mathrm{med}}=\mathrm{median}\left\{r,g,b\right\}$;4:**if** $u_{p_{\mathrm{med}}}^{T}p\leq 1$ **then**5: $q_{\alpha }=\frac{1}{l-p_{\mathrm{med}}}p$;6:**else**7: $q_{\alpha }=1+\frac{1}{2-(l-p_{\mathrm{med}})}\left(p-1\right)$;8:**end if**9:$\tilde{p}=\phi^{NM}\left(q_{\alpha },\tilde{l}\right)$;

## 4. Experimental Results

In this section, we show the experimental results of hue-preserving color image enhancement with Naik and Murthy’s method [2], Yang and Lee’s method [9] and the proposed method.

**Figure 6:**
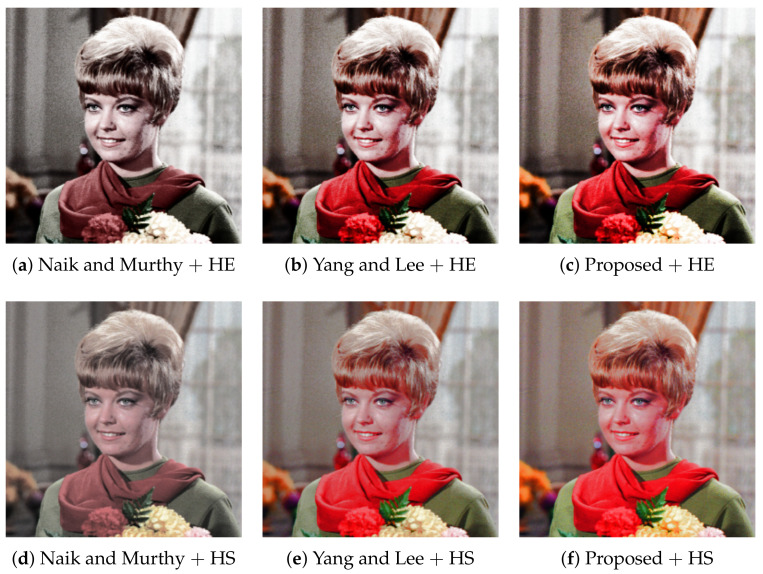
Hue-preserving color image enhancement: The top row (**a**–**c**) shows the output images with histogram equalization (HE) for computing target intensities, and the bottom row (**d**–**f**) shows that with histogram specification (HS). The left (**a**,**d**), middle (**b**,**e**) and right (**c**,**f**) columns show the results with Naik and Murthy’s method, Yang and Lee’s method and the proposed methods, respectively.

Figure 6 shows six enhanced color images computed from the common input image in Figure 2a. In Figure 6a, the contrast is enhanced by HE, compared with that of the original image, and the gamut problem is avoided by Naik and Murthy’s method successfully. However, the saturation is deteriorated as shown in Section 3.1. In Figure 6b, Yang and Lee’s method recovers the saturation better than that of Naik and Murthy. In Figure 6c, the proposed method improves the saturation further than that of Yang and Lee. This result coincides with the results of Yu’s method [10]. Figure 6d shows the result of HS combined with Naik and Murthy’s method, where the contrast is suppressed while the saturation is improved, compared with Figure 6a. Figure 6e of Yang and Lee’s method shows a more colorful image than Figure 6d. Figure 6d,e coincides with the results of Inoue’s method [15]. Figure 6f of the proposed method shows further improved saturation than Figure 6e, specifically for the regions of the facial skin and curtain in the background.

Table 1 shows the mean saturation of each image in Figure 6, which numerically confirms that the proposed method achieved maximum saturation among the compared methods for both HE and HS.

**Figure 7:**
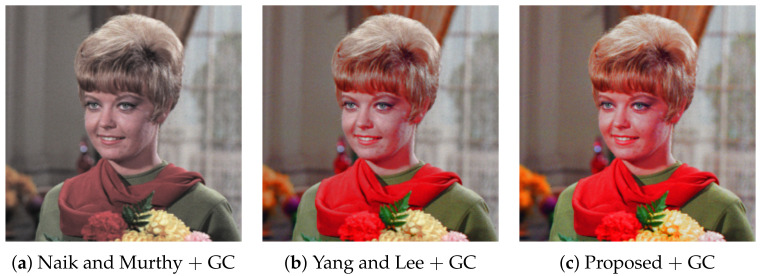
Gamma correction: the intensity of the input image in Figure 2a is modified by the gamma correction with γ=0.5, which is adjusted manually. Then, the corresponding RGB color values are determined by Naik and Murthy’s method (**a**), Yang and Lee’s method (**b**) and the proposed methods (**c**).

As well as the compared methods, the proposed method is also applicable to other intensity transformation methods. For example, we show the results of the gamma correction (GC) in Figure 7, where we choose γ=0.5 to obtain subjectively better results.

**Figure 8:**
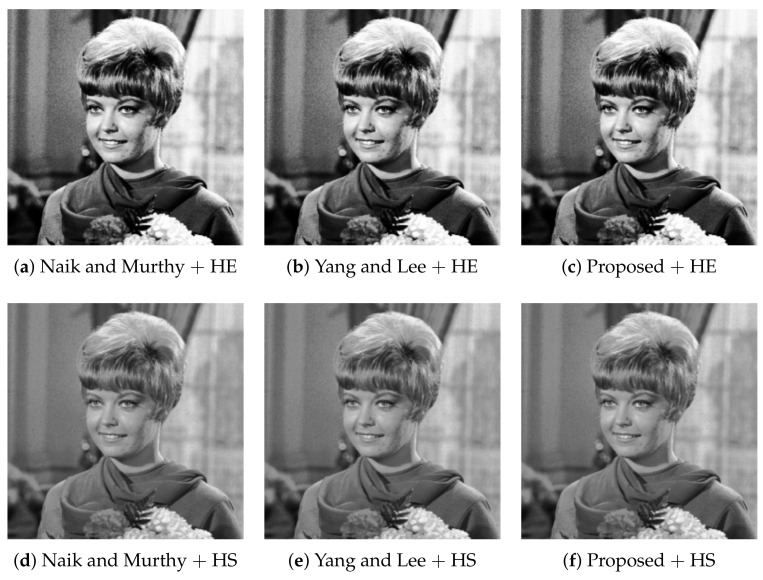
Luminance images computed from the corresponding color images in Figure 6: the top row (**a**–**c**) shows the luminance images with histogram equalization (HE) for computing target intensities, and the bottom row (**d**–**f**) shows that with histogram specification (HS).

The compared three methods preserve the intensity of every input color image as shown in Figure 8, where we can see the luminance [9] images, which are the normalized intensity images, as $l_{ij}/3=\left(r_{ij}+g_{ij}+b_{ij}\right)/3$, corresponding to the enhanced color images in Figure 6. The intensity images in the top row are identical to the histogram equalized-grayscale image in Figure 3b, and those in the bottom row are identical to the histogram specified-grayscale image in Figure 3c.

**Figure 9:**
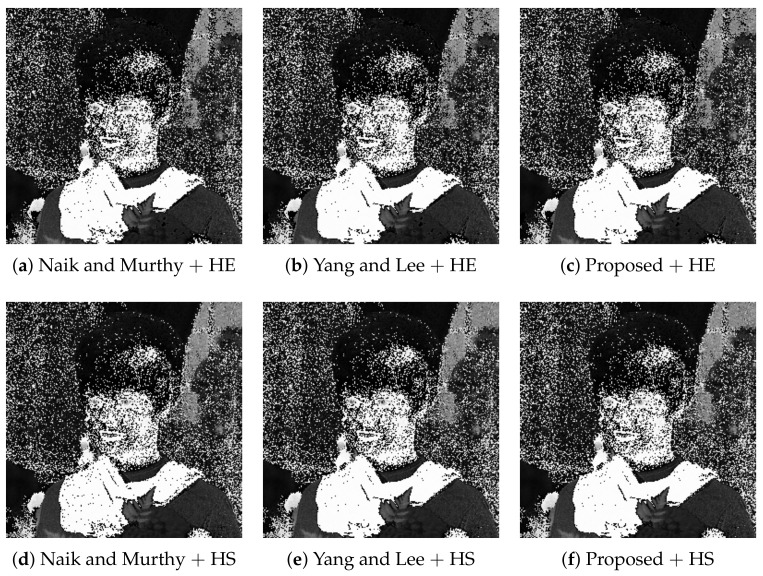
Hue images computed from the corresponding color images in Figure 6: the top row (**a**–**c**) shows the luminance images with histogram equalization (HE) for computing target intensities, and the bottom row (**d**–**f**) shows that with histogram specification (HS).

**Figure 10:**
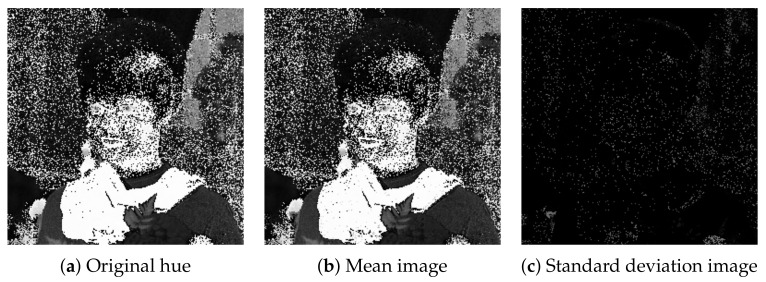
Mean and standard deviation of hue values of the six images in Figure 9 compared with the original hue image (**a**) computed from the input color image in Figure 2a: (**b**) the mean image of Figure 9a–f, and (**c**) their standard deviation.

We also computed hue images from the color images in Figure 6 as shown in Figure 9, where each hue value is computed from the RGB color values on the basis of the definition of hue given by the Equation (6.2–2) in the reference [12]. All images in Figure 9 are similar to the original hue image shown in Figure 10a. We observed that the hue values contain numerical errors whose maximum standard deviation is 0.50 for normalized RGB color vectors. The mean and standard deviation of hue values at every pixel are visualized in Figure 10b,c, respectively. In Figure 10c, the black and white pixels indicate the standard deviation values 0 and 1, respectively.

**Figure 11:**
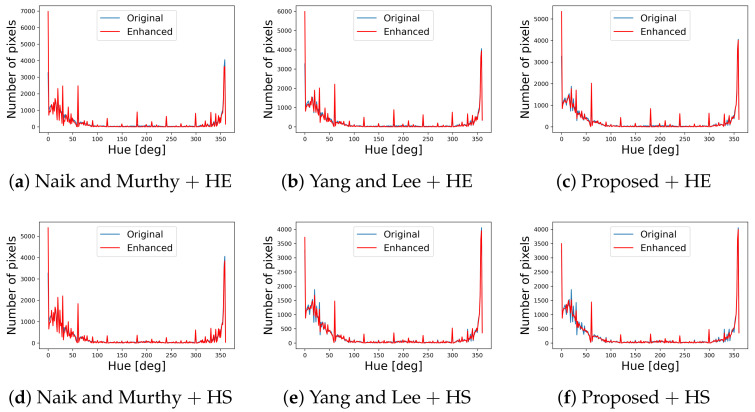
Hue histograms computed from the color images in Figure 6. In each graph, the blue line denotes the hue histogram of the original color image in Figure 2a, and the red line denotes that of the enhanced image by the corresponding method indicated in the subcaption.

Figure 11 shows hue histograms of the color images in Figure 6, where the vertical and horizontal axes of each graph denote the number of pixels and hue in degrees, respectively. The blue and red lines denote the original enhanced histograms, which are similar to each other. The small differences between them are mainly resulted from the quantization errors caused by recording color values in an image file format.

**Figure 12:**
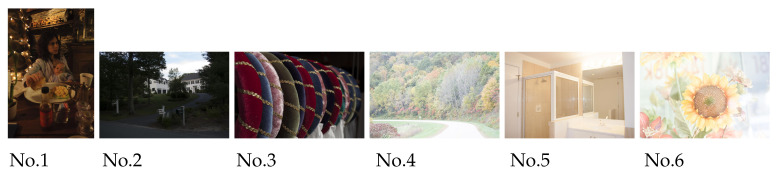
Color images selected from Afifi’s image dataset [17], which was originally rendered using raw images taken from the MIT-Adobe FiveK dataset [18]. Nos. 1 to 3 are underexposed images, and Nos. 4 to 6 are overexposed ones.

Next, Figure 12 shows six color images selected from an image dataset by Afifi et al. [17], where the first three images are underexposed, and the last three ones are overexposed. We selected the images in Figure 12 based on their relative exposure values (EVs), that is, the underexposed images have smaller than or equal to −1 relative EVs, and the overexposed images have greater than or equal to +1 relative EVs. As the dataset was originally rendered using raw images taken from the MIT-Adobe FiveK dataset, their dataset follows the original license of the MIT-Adobe FiveK dataset [18].

**Figure 13:**
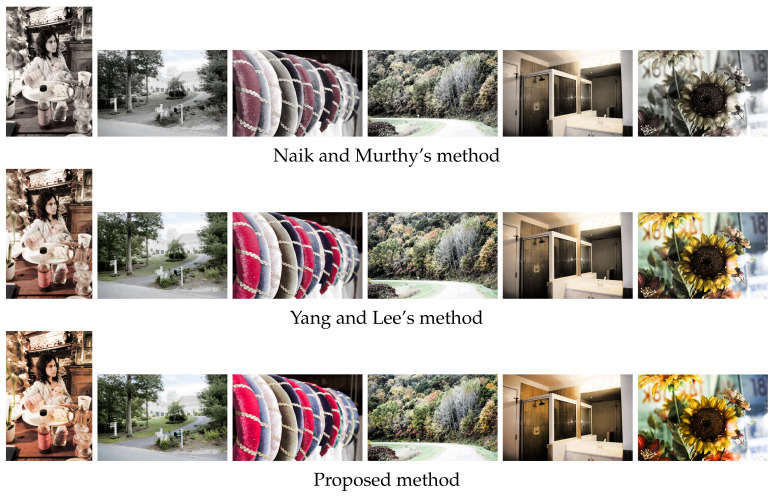
Histogram equalization applied to color images in Figure 12, where the top, middle and bottom rows show the output images by Naik and Murthy’s method, Yang and Lee’s method and the proposed method, respectively.

Figure 13 shows the results of HE applied to the images in Figure 12, where Naik and Murthy’s method in the top row enhances the contrast without a gamut problem for both under- and overexposed images; however, the resultant images look like sepia or grayscale ones with faded colors. Yang and Lee’s method in the middle row recovers the color saturation compared with the above results. The proposed method in the bottom row improves the saturation further than the above two results.

**Figure 14:**
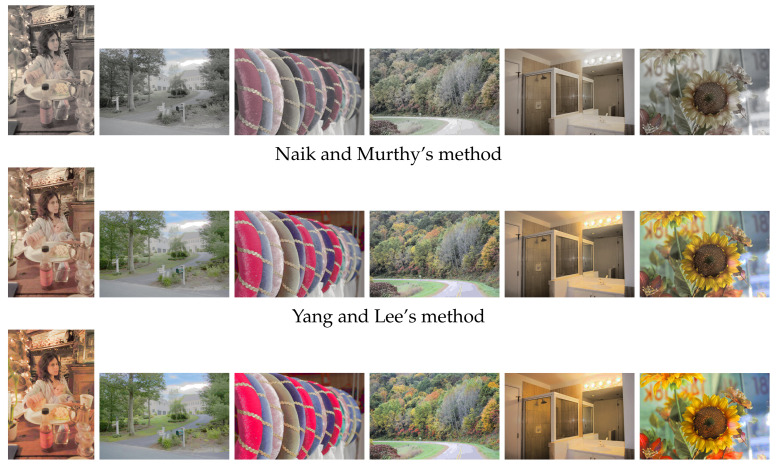
Histogram specification applied to color images in Figure 12, where the top, middle and bottom rows show the output images by Naik and Murthy’s method, Yang and Lee’s method and the proposed method, respectively.

Figure 14 also shows the results of HS applied to the images in Figure 12, where we obtained more natural results of contrast enhancement than the results of HE in Figure 13.

**Figure 15:**
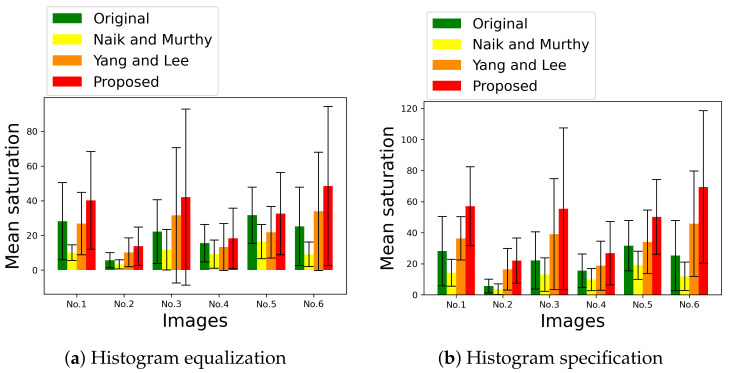
Mean saturation of enhanced images in Figure 13a and Figure 14b, where the vertical axes denote the mean saturation, and the horizontal axes denote the image ID numbered in Figure 12. The yellow, orange and red bars denote Naik and Murthy’s method, Yang and Lee’s method and the proposed method, respectively. The error bars denote the standard deviation of saturation.

Figure 15 shows two graphs, (**a**) and (**b**), of the mean saturation of the above images in Figure 13 and Figure 14 enhanced by HE and HS, respectively, where the vertical and horizontal axes of each graph denote the mean saturation and the images numbered from 1 to 6 in Figure 12. The green, yellow, orange and red bars denote the original image in Figure 2a, Naik and Murthy’s method, Yang and Lee’s method and the proposed method, respectively, which show that Naik and Murthy’s method decreases the mean saturation from the original values, Yang and Lee’s method outperforms that of Naik and Murthy, and the proposed method outperforms that of Yang and Lee, quantitatively. In Figure 15, the standard deviation of saturation in each image is also shown with an error bar, where the longer the error bar becomes, the more various saturation values the enhanced image contains.

Table 2 summarizes the mean saturation of the images in Figure 13 and Figure 14 obtained by the compared methods, where the proposed method achieves the maximum values among the compared methods for both HE and HS.

Similarly, Table 3 summarizes the standard deviation of saturation for all compared methods, where the proposed method achieves the maximum values among the compared methods for both HE and HS.

**Figure 16:**
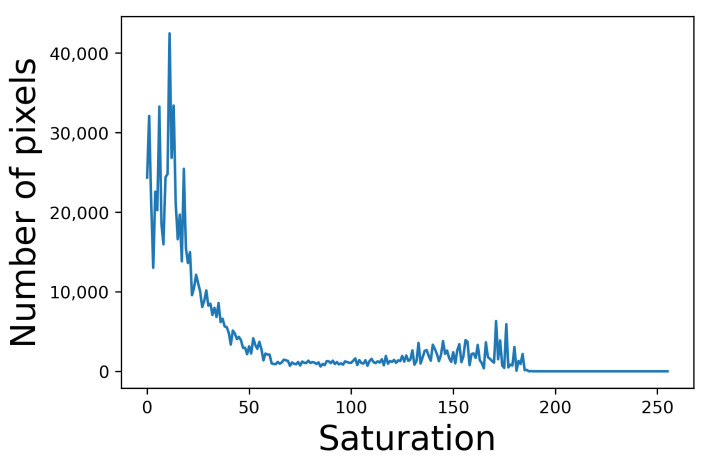
Saturation histogram of image No. 3 enhanced by the proposed method with HE.

In Figure 15, we find that the standard deviation of saturation in an image can be larger than the mean saturation. For example, we show the histogram of saturation of image No. 3 enhanced by the proposed method with HE in Figure 16, where the mean saturation is 42.1 and the standard deviation is 50.8. All values of the saturation are included in the range [0,195.2].

**Figure 17:**
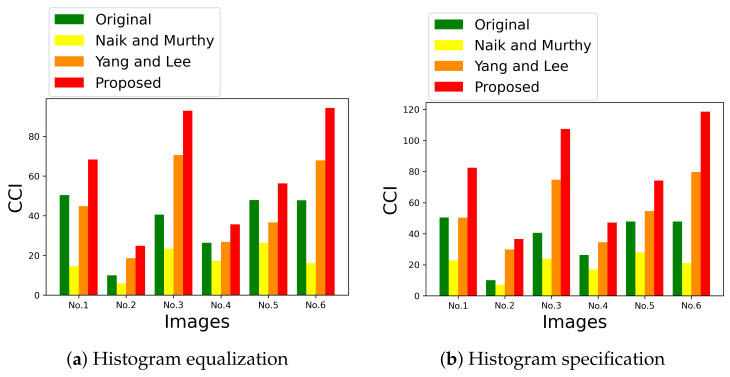
Color colorfulness index (CCI) [4]: the intensities are transformed by histogram equalization (**a**) and histogram specification (**b**).

We also evaluated the performance with the color colorfulness index (CCI) defined in (10) in [4] as shown in Figure 17, where the vertical and horizontal axes denote the CCI value and image numbers, respectively. The value of CCI varies from 0 (achromatic image) to the maximum (most colorful image) [4]. For both HE (**a**) and HS (**b**), the proposed method denoted by red bars achieves the highest values among the compared methods.

**Figure 18:**
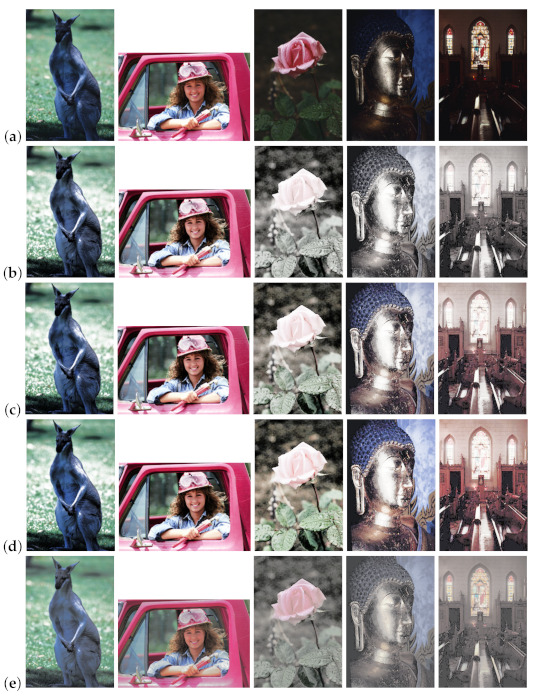

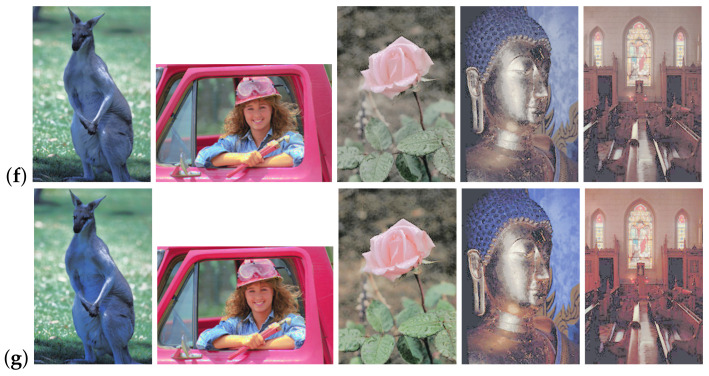
Assembled result: (**a**) original images, (**b**) Naik and Murthy’s method + HE, (**c**) Yang and Lee’s method + HE, (**d**) proposed method + HE, (**e**) Naik and Murthy’s method + HS, (**f**) Yang and Lee’s method + HS, (**g**) proposed method + HS.

Finally, Figure 18 shows an assembled result for browsing the performance of compared methods, where five original images in row (**a**) are enhanced by six different combinations of compared methods to obtain enhanced images listed in rows, (**b**) to (**g**). These results also support the effectiveness of the proposed method visually. The rows (**b**), (**c**) and (**d**) show the enhanced images by Naik and Murthy’s method, Yang and Lee’s method and the proposed method with HE; the rows (**e**), (**f**) and (**g**) show that with HS. These results exemplify that HE enhances the contrast strongly, and HS used in this paper enhances the saturation dominantly.

## 5. Discussion

The above experimental results demonstrate the validity of the theoretical properties of the compared methods, i.e., Naik and Murthy’s method gives faded colors, Yang and Lee’s method recovers the weak point of Naik and Murthy’s method, and the proposed method improves the color saturation further than that of Yang and Lee by eliminating the middle part of their method where they directly use Naik and Murthy’s method, which generates faded colors almost every time.

For generating the target intensity image required for color image enhancement, we compared HE and HS in the above experiments, where we observed that HE frequently enhanced the contrast excessively. On the other hand, the adopted RGB color cube-based HS achieved moderate enhancement results in a parameter-free manner as well as the conventional HE. Therefore, we would like to recommend its adoption as an alternative to HE for contrast enhancement.

In this paper, we used a conventional method for HE and HS that is based on the cumulative histograms. On the other hand, Nikolova and Steidl have proposed a more sophisticated method for exact HS [19,20], which can be substituted for the conventional one to produce better results of color image enhancement in the proposed method.

## 6. Conclusions

In this paper, we proposed a method for hue-preserving color image enhancement in an RGB color cube, and compared it with two representative methods in this context: those of Naik and Murthy, and Yang and Lee. It is theoretically confirmed that the proposed method can improve the saturation better than those methods. The experimental results also demonstrated the effectiveness of the proposed method, compared with them, visually and numerically.

## Figures and Tables

**Table 1:** Mean saturation of the images in Figure 6, where the maximum value in each row is indicated in boldface.

	Naik and Murthy [2]	Yang and Lee [9]	Proposed
Histogram equalization	13.73	27.40	**33.39**
Histogram specification	15.12	39.98	**49.89**

**Table 2:** Mean saturation, where the maximum value in each row is indicated in boldface.

	Naik and Murthy [2]	Yang and Lee [9]	Proposed
Histogram equalization	9.94	22.93	**32.55**
Histogram specification	11.91	31.74	**46.81**

**Table 3:** Standard deviation of saturation, where the maximum value in each row is indicated in boldface.

	Naik and Murthy [2]	Yang and Lee [9]	Proposed
Histogram equalization	7.31	21.30	**29.52**
Histogram specification	8.03	22.20	**30.93**

## Data Availability

Not applicable.

## Abbreviations

The following abbreviations are used in this manuscript:

RGB	Red, green and blue
HSI	Hue, saturation and intensity
HE	Histogram equalization
HS	Histogram specification
HM	Histogram manipulation
CCI	Color colorfulness index
GC	Gamma correction
